# Detection of Early-Stage Degeneration in Human Articular Cartilage by Multiparametric MR Imaging Mapping of Tissue Functionality

**DOI:** 10.1038/s41598-019-42543-w

**Published:** 2019-04-11

**Authors:** Sven Nebelung, Manuel Post, Matthias Knobe, Markus Tingart, Pieter Emans, Johannes Thüring, Christiane Kuhl, Daniel Truhn

**Affiliations:** 1 Department of Diagnostic and Interventional Radiology, Aachen University Hospital, Aachen, Germany; 2 Department of Trauma Surgery, Aachen University Hospital, Aachen, Germany; 3 Department of Orthopaedics, Aachen University Hospital, Aachen, Germany; 4 Department of Orthopaedic Surgery, Maastricht University Medical Center, Maastricht, The Netherlands; 5 Institute of Imaging and Computer Vision, RWTH Aachen, Aachen, Germany

## Abstract

To assess human articular cartilage tissue functionality by serial multiparametric quantitative MRI (qMRI) mapping as a function of histological degeneration. Forty-nine cartilage samples obtained during total knee replacement surgeries were placed in a standardized artificial knee joint within an MRI-compatible compressive loading device and imaged *in situ* and at three loading positions, i.e. unloaded, at 2.5 mm displacement (20% body weight [BW]) and at 5 mm displacement (110% BW). Using a clinical 3.0 T MRI system (Achieva, Philips), serial T1, T1ρ, T2 and T2* maps were generated for each sample and loading position. Histology (Mankin scoring) and biomechanics (Young’s modulus) served as references. Samples were dichotomized as intact (*int*, n = 27) or early degenerative (*deg*, n = 22) based on histology and analyzed using repeated-measures ANOVA and unpaired Student’s t-tests after log-transformation. For T1ρ, T2 and T2*, significant loading-induced differences were found in *deg* (in contrast to *int*) samples, while for T1 significant decreases in all zones were observed, irrespective of degeneration. In conclusion, cartilage functionality may be visualized using serial qMRI parameter mapping and the response-to-loading patterns are associated with histological degeneration. Hence, loading-induced changes in qMRI parameter maps provide promising surrogate parameters of tissue functionality and status in health and disease.

## Introduction

While osteoarthritis (OA) involves the entire joint, its central hallmark is the progressive degeneration of articular cartilage. Early cartilage degeneration is considered to be reversible as long as preventive interventions (i.e. pharmacotherapy, lifestyle modification or axis-modifying surgery) are still successful^1^. As such, cartilage degeneration needs to be reliably detected at the earliest stages, which is currently not possible using clinical routine imaging modalities^2,3^. Quantitative MRI (qMRI) techniques such as T2 and T1ρ mapping have therefore received considerable scientific attention^4,5^. QMRI techniques are guided by the prospect of more standardized and objective tissue assessment and assess alterations of the extracellular matrix (ECM) constituents^2,6^, thereby providing quantitative information on composition (beyond structure). However, these techniques’ considerable inter- and intra-individual variability makes differentiation of early-to-moderate stages of degeneration challenging^5,7,8^, as these are characterized by only minor alterations in structure and composition^9^. Among other changes^2^, cartilage degeneration is characterized by gradual reductions in mechanical stiffness throughout the entire sample depth^10^, resulting in increasing local strains (under constant stress) with progressive degeneration^9^. Hence, biomechanical stimuli have been implemented within MRI scan protocols to assess cartilage tissue functionality: Recent approaches have used qMRI parameter maps to quantify the tissue’s response to loading with promising results^11–17^; yet, consistent referencing, for example to histology, has seldom been performed. To our mind, histological referencing as the current reference standard is obligatory when assessing cartilage functionality as a potential marker of (early) degeneration. Moreover, tissue dynamics have been investigated primarily *ex vivo* using samples or joint surfaces^13,14,18,19^; however, studying cartilage functionality in a whole-joint configuration seems beneficial to obtain physiologically meaningful results^16,20^. In earlier studies by our group and others, the physiological response-to-loading patterns of histologically intact cartilage as a surrogate parameter of cartilage functionality were defined^19^. Moreover, the structural and compositional correlates of T2 relaxation relevant to functionality were defined in intact cartilage based on an anisotropic hyperelastic constitutive tissue model^21^. Other studies investigated cartilage functionality and its alterations in degenerative joint disease. When assessing loading-induced changes in cartilage in the presence of degeneration, larger reductions in T1ρ and T2 were found OA knees than in non-OA knees (as determined radiographically), indicating altered load-bearing in OA^16^. It is against this background that this study’s purpose was to assess cartilage functionality as a function of histological degeneration using multiparametric qMRI techniques and an MRI-compatible whole-knee joint loading device for the *in-situ* assessment of chondral samples. Details on the device’s development, construction and validation were reported previously^22^. Hence, loading-induced changes in intact (*int*) and early degenerative (*deg*) human articular cartilage samples were studied using serial T1, T1ρ, T2 and T2* mapping and subsequently referenced both histologically and biomechanically to identify degeneration-dependent differences in loading patterns. We hypothesized that qualitative and quantitative loading-induced intra-tissue changes are related to tissue degeneration and may be used to improve the diagnostic accuracy of the qMRI parameters investigated.

## Results

**Figure 1:**
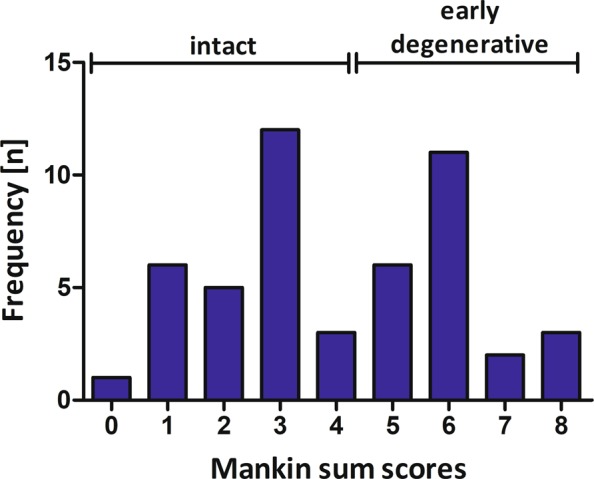
Distribution of Mankin sum scores. After sample dichotomization, 27 samples constituted the intact group and 22 samples the early degenerative group.

All 49 samples underwent complete MR imaging and biomechanical as well as histological assessment. Figure 1 gives a graphical illustration of the different Mankin sum scores (MSS). After sample dichotomization, 27 samples constituted the intact (*int*) group and 22 samples the degenerative (*deg*) group. Consequently, significant group-wise differences were found in MSS (*int* vs. *deg*: 2.4 ± 1.1 vs. 6.0 ± 0.9; p < 0.001) and Young’s Modulus (YM) (0.6 ± 0.3 vs. 0.4 ± 0.3 [MPa]; p = 0.003) (Table 1). Additional details of the histological, biomechanical and unloaded qMRI parameter values are given in Table 1. In the unloaded configuration, significant differences between *int* and *deg* samples were only found for T1 and T2, where *int* samples displayed significantly lower values than *deg* samples, however, only in the deep tissue zones (T1 [dp]: p = 0.029; T2 [dp]: p = 0.008).

**Table 1:** MR imaging, histological and biomechanical parameters of human cartilage samples.

		Int (n = 27)		Deg (n = 22)		p-values	All (n = 49)	
		M ± SD	Range	M ± SD	Range		M ± SD	Range
**T1**	entire cartilage sample (ECS)	734.5 ± 121.3	555.0–1112.0	777.0 ± 104.0	585.3–988.2	ns	753.6 ± 114.7	555.0–1112.0
	superficial zone (sf)	784.1 ± 133.8		788.3 ± 100.3		ns	786.0 ± 118.8	
	deep zone (dp)	684.4 ± 121.2		763.3 ± 123.4		**0.029**	719.9 ± 127.2	
**T1ρ**	ECS	101.5 ± 16.8	69.0–149.4	98.4 ± 16.2	71.7–150.6	ns	100.2 ± 16.4	69.0–150.6
	sf	108.5 ± 18.7		103.7 ± 18.2		ns	106.6 ± 18.4	
	dp	94.9 ± 18.1		93.0 ± 17.7		ns	94.1 ± 17.7	
**T2**	ECS	40.8 ± 5.4	33.3–56.1	43.1 ± 7.8	32.3–63.6	ns	41.8 ± 6.6	32.3–63.6
	sf	46.4 ± 7.0		46.3 ± 9.9		ns	46.3 ± 8.3	
	dp	35.1 ± 5.8		39.9 ± 7.3		**0.008**	37.3 ± 6.9	
T2*	ECS	22.0 ± 4.3	13.6–31.5	22.0 ± 4.3	15.5–32.0	ns	22.0 ± 4.2	13.6–32.0
	sf	23.9 ± 5.2		22.7 ± 5.1		ns	23.4 ± 5.2	
	dp	19.9 ± 4.3		21.7 ± 6.0		ns	20.7 ± 5.1	
**Mankin Sum Score**		2.4 ± 1.1	0.0–4.0	6.0 ± 0.9	5.0–8.0	**<0.001**	4.0 ± 2.1	0.0–8.0
**Young’s Modulus [MPa]**		0.55 ± 0.31	0.19–1.49	0.35 ± 0.34	0.02–1.58	**0.003**	0.46 ± 0.33	0.02–1.58

Data are given as Mean ± Standard Deviation (M ± SD) or range (minimum – maximum), respectively. P-values indicate, whether group-wise comparisons of histologically intact (*int*) vs. early degenerative (*deg*) samples based on the unpaired Student’s t-test after log-transformation were significant or not (ns). Significant group-wise differences are given in bold-type. Units of quantitative MRI parameters: ms. Units of Young’s Modulus: MPa.

Distinct qualitative and quantitative changes were observed with loading: First, mean sample height was significantly reduced (Δ_2.5_: −6.1 ± 22.0%; Δ_5.0_: −15.6 ± 25.8%; p < 0.001), while mean sample width remained about constant (Δ_2.5_: 1.9 ± 7.8%; Δ_5.0_: 3.1 ± 11.1%; p = 0.127). Correspondingly, the number of detected pixels significantly decreased in all zones (entire cartilage sample [ECS]: 108 ± 34 [δ_0_], 97 ± 28 [δ_2.5_], 83 ± 25 [δ_5.0_], p < 0.001; superficial zone [sf]: 54 ± 18 [δ_0_], 48 ± 14 [δ_2.5_], 40 ± 14 [δ_5.0_], p < 0.001; deep zone [dp]: 54 ± 18 [δ_0_], 48 ± 15 [δ_2.5_], 40 ± 14 [δ_5.0_], p < 0.001). Second, distinct loading-induced changes were found in absolute qMRI parameter values (Table 2 and Supplementary Table S1): For T1, consistent significant decreases were found in all tissue zones, irrespective of degeneration. For T1ρ, significant increases were observed in *deg* samples’ deep zones only. This increase in *deg* samples rendered T1ρ changes over all samples significant, too. In *int* samples, however, T1ρ changes were inconsistent. For T2, significant decreases were found in the superficial zone and corresponding increases in the deep zone. Although these changes were significant in all regions-of-interest (ROIs) of *int* samples and only in the deep zone of *deg* samples, they were more pronounced in *deg* samples. For T2*, changes were equally more pronounced and significant in *deg* samples only.

**Table 2:** Absolute qMRI parameter values in response to displacement-controlled compressive loading [ms].

		T1				T1ρ				T2				T2*			
		δ_0_	δ_2.5_	δ_5.0_	p-value	δ_0_	δ_2.5_	δ_5.0_	p-value	δ_0_	δ_2.5_	δ_5.0_	p-value	δ_0_	δ_2.5_	δ_5.0_	p-value
all (n = 49)	ECS	753.6 ±114.7	700.6 ±117.4	667.3 ±145.3	**<0.001(1)**	99.6 ±16.2	106.1 ±21.0	108.0 ±24.1	0.181	41.8 ±6.6	43.1 ±8.3	42.0 ±8.9	0.138	22.0 ±4.2	22.6 ±4.9	22.9 ±5.1	0.210
	sf	786.0 ±118.8	699.1 ±125.0	660.0 ±144.9	**<0.001(2)**	106.6 ±18.4	106.5 ±23.9	107.3 ±25.3	0.952	46.3 ±8.3	45.9 ±9.3	42.4 ±8.7	**<0.001(11)**	23.4 ±5.2	24.8 ±7.0	24.4 ±5.9	0.172
	dp	719.9 ±127.2	698.3 ±118.2	679.9 ±161.0	**<0.001(3)**	94.1 ±17.7	108.4 ±21.9	110.8 ±26.0	**<0.001(9)**	37.3 ±6.9	40.3 ±9.4	41.3 ±10.2	**<0.001(12)**	20.7 ±5.1	20.1 ±4.1	21.0 ±4.8	0.323
int (n = 27)	ECS	734.5 ±121.3	677.0 ±116.6	638.2 ±144.8	**<0.001(4)**	100.4 ±16.5	106.1 ±21.8	104.8 ±23.8	0.696	40.8 ±5.4	42.6 ±7.3	39.7 ±7.4	**0.002(13)**	22.0 ±4.3	22.6 ±4.8	22.0 ±4.4	0.565
	sf	784.1 ±133.8	685.4 ±124.8	640.0 ±148.8	**<0.001(5)**	108.5 ±18.7	107.0 ±22.4	104.0 ±25.2	0.491	46.4 ±7.0	45.7 ±7.6	41.2 ±7.6	**<0.001(14)**	23.9 ±5.2	24.6 ±6.4	23.8 ±5.4	0.798
	dp	684.4 ±121.2	663.9 ±115.9	642.2 ±159.0	**0.003(6)**	94.9 ±18.1	105.5 ±21.9	108.1 ±25.3	0.118	35.1 ±5.8	38.8 ±8.6	37.9 ±8.2	**<0.001(15)**	19.9 ±4.3	20.2 ±4.4	20.1 ±4.3	0.815
deg (n = 22)	ECS	777.0 ±104.0	729.7 ±114.2	703.1 ±140.9	**<0.001(7)**	98.4 ±16.2	106.2 ±20.6	112.1 ±24.7	0.093	43.1 ±7.8	43.8 ±9.5	44.8 ±10.0	0.443	22.0 ±4.3	22.4 ±5.2	24.1 ±5.7	**0.029(17)**
	sf	788.3 ±100.3	716.0 ±126.1	684.6 ±139.4	**<0.001(8)**	103.7 ±18.2	105.7 ±26.7	112.1 ±25.4	0.506	46.3 ±9.9	46.0 ±11.2	43.8 ±10.0	0.115	22.7 ±5.1	25.1 ±7.8	25.1 ±6.5	0.0504
	dp	763.3 ±123.4	740.5 ±109.3	726.2 ±154.5	0.062	93.0 ±17.7	112.0 ±21.9	114.2 ±27.3	**0.003(10)**	39.9 ±7.3	42.0 ±10.2	45.4 ±10.9	**0.002(16)**	21.7 ±6.0	20.0 ±3.9	22.1 ±5.3	0.076

Segmentation included the entire cartilage sample (ECS) as well as superficial (sf) and deep (dp) zones. Upon log-transformation, repeated measures ANOVA was used to detect differences between δ0 (unloaded), δ2.5 (2.5 mm displacement) and δ5.0 (5.0 mm displacement). Data are mean ± standard deviation [ms] and p-value. Significant differences are displayed in bold-type followed by consecutive numbers indicating post-test details (see Supplementary Table S1). Abbreviations as in Table 1.

Best sensitivities to differentiate *int* from *deg* samples were found for T1ρ and T2 (unloaded) and for T2* Δ_5.0_ (loaded). Highest combined sensitivities were found for T2 - T2*Δ_5.0_ and T1ρ - T2*Δ_5.0_, while highest combined specificities were found for T2 - T2Δ_2.5_ and T1 - T2Δ_2.5_ (Table 3

**Table 3:** Sensitivities and specificities of individual quantitative MRI parameters [ms], relative changes in quantitative MRI parameters [%] and selected combinations thereof.

	Quantitative MRI parameters				Relative changes in quantitative MRI parameters								Selected combinations							
	T1	T1ρ	T2	T2*	T1Δ2.5	T1Δ5.0	T1ρΔ2.5	T1ρΔ5.0	T2Δ2.5	T2Δ5.0	T2*Δ2.5	T2*Δ5.0	T2 +T2*Δ5.0	T1ρ +T2*Δ5.0	T2 +T2Δ2.5 (*)	T1 +T2Δ2.5 (*)	T1 +T1Δ2.5	T1ρ +T1ρΔ2.5	T2 +T2Δ2.5	T2* +T2*Δ2.5
Sensitivity	0.630	0.810	0.852	0.630	0.741	0.741	0.667	0.619	0.704	0.667	0.593	0.778	0.967	0.958	0.600	0.444	0.904	0.937	0.956	0.849
Specificity	0.364	0.313	0.364	0.273	0.227	0.182	0.250	0.188	0.500	0.364	0.409	0.409	0.149	0.128	0.682	0.682	0.083	0.078	0.182	0.112
int [range, min; max]	638.9; 868.3	83.8; 116.6	35.2; 48.4	17.8; 26.2	−17.0; 1.9	−26.2; −0.3	−18.0; 33.3	−20.3; 32.6	−4.2; 12.6	−15.2; 9.8	−15.2; 9.9	−16.7; 20.7	n/a	n/a	n/a	n/a	n/a	n/a	n/a	n/a
deg [range, min; max]	<638.9; >868.3	<83.8; >116.6	<35.2; >48.4	<17.8; >26.2	<−17.0; >1.9	<−26.2; >−0.3	<−18.0; >33.3	<−20.3; >32.6	<−4.2; >12.6	<−15.2; >9.8	<−15.2; >9.9	<−16.7; >20.7	n/a	n/a	n/a	n/a	n/a	n/a	n/a	n/a

Combined maximum sensitivities were calculated according to the believe the positive rule, while (*) indicates maximum specificity based on the believe the negative rule. Abbreviations as in Table 1.

).

Group-wise comparisons of relative changes in qMRI parameters did not reveal significant differences (Supplementary Table S2). Similarly, no significant correlations were found between relative changes in qMRI parameters and YM or MSS (Supplementary Table S3).

**Figure 2:**
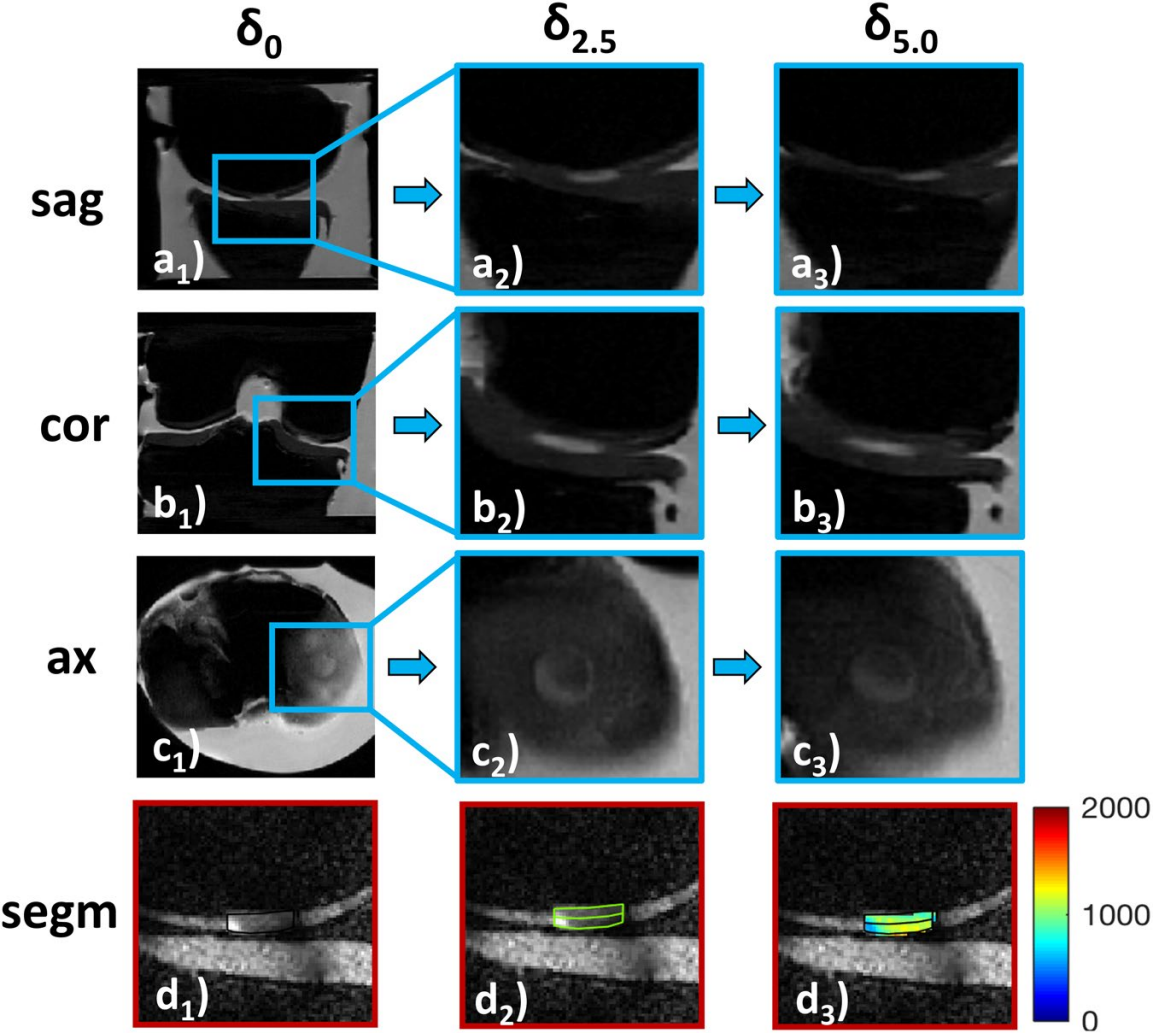
Serial morphological images as a function of loading. Proton density-weighted images obtained in the sagittal (*sag*; **a**), coronal (*cor*; **b**) and axial (*ax*; **c**) orientation demonstrating the wirosil® silicone-covered femur (top in a and b) and tibia (bottom in **a** and **b**). Consecutive displacement positions are displayed: unloaded, δ_0_ (a_1_–c_1_); at 2.5 mm displacement, δ_2.5_ (a_2_–c_2_); at 5.0 mm displacement δ_5.0_ (a_3_–c_3_). The native cartilage sample (hyperintense) is positioned within the standardized defect at the medial femoral condyle surrounded by silicone (hypointense) (framed in blue [a_1_–c_1_] and displayed at higher magnifications [a_2,3_–c_2,3_]). Exemplary segmentation outlines and regions-of-interest (*segm*, **d**). Upon segmentation of the entire sample outline (black in d_1_), regions-of-interest were defined by equally partitioning the sample cross-sectional area into the superficial (bottom in d_2_ and d_3_) and deep cartilage zones (top in d_2_ and d_3_). Color-coded T1-maps overlaid onto the morphological T1 image (d_3_). Scale bars extend from 0–2000 ms. Mid-coronal image (**d**).

**Figure 3:**
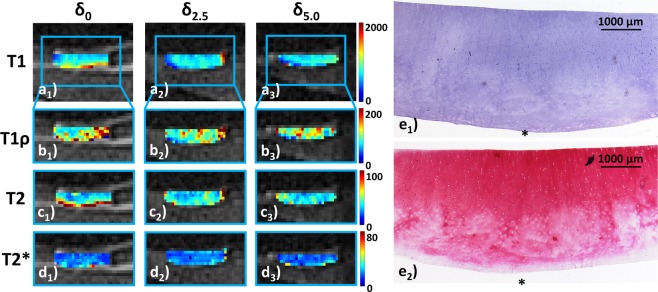
Serial qMRI parameter maps under loading and corresponding histological sections of intact cartilage. QMRI parameter maps (T1 [**a**]; T1ρ [**b**]; T2 [**c**]; T2* [**d**]) are displayed in the unloaded configuration δ_0_ (a_1_–d_1_) and at consecutive loading positions δ_2.5_ (a_2_–d_2_) and δ_5.0_ (a_3_–d_3_). T1ρ, T2 and T2* maps are displayed at higher magnification for better visualization ((**b**–**d**) framed in blue). In this sample, relatively homogeneous qMRI parameter distributions at δ_0_ remained largely unaltered at δ_2.5_ and δ_5.0_. Only for T1ρ, pre-existent slight focal signal heterogeneities changed a bit with loading. The first morphological image obtained of each series was used for qMRI parameter overlays. Entire sample width is 8 mm. Corresponding histological sections revealed the absence of substantial structural surface or sub-surface alterations, while focal cell proliferation (only visible at higher magnification [not shown]) and moderate discoloration on proteoglycan staining were found. Hematoxylin/eosin (e_1_) and Safranin O staining (e_2_). MSS 3. Histological sections are displayed bottom-down in keeping with the orientation of the serial qMRI maps; hence, asterisks indicate the tissue’s surface.

**Figure 4:**
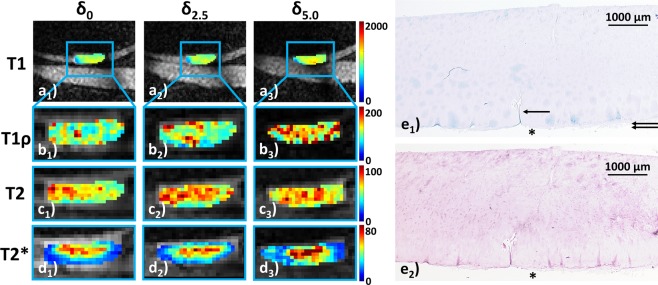
Serial qMRI parameter maps under loading and corresponding histological sections of early degenerative cartilage. For all qMRI parameters, loading-induced increases were observed. Irregular signal hyperintensities (at δ_0_) became more diffuse and widespread to eventually involve the majority of the sample’s cross-sectional area (in particular T1ρ and T2). Histologically, superficial clefts (indicated by single arrow in e_1_) and pannus formation (indicated by double arrows in e_1_) were found alongside diffuse hypercellularity (only visible at higher magnification [not shown]) and severe discoloration on proteoglycan staining. MSS 8. Image details as in Fig. 3.

Morphologically, sample width and signal intensity in PDW images remained grossly unaltered, while sample height consistently decreased (Fig. 2a–c). Serial qMRI parameter maps were reflective of the quantitative changes outlined above: While in *int* samples, relatively homogeneous parameter distributions at δ_0_ were found that remained largely unaltered at δ_2.5_ and δ_5.0_ (Fig. 3), *deg* samples displayed inconsistent loading-induced changes: In some samples, diffuse signal alterations became more widespread to eventually involve the majority of the sample cross-sectional area (Fig. 4), while in other samples focal signal alterations were less distinct to nearly disappear altogether (Supplementary Fig. S1).

## Discussion

The most important finding of this study is that loading-induced changes in some serial qMRI parameter maps are related to histological degeneration and improve their diagnostic accuracy, which provides a solid scientific framework to assess cartilage functionality in future applications.

As demonstrated previously^22^, the MRI-compatible whole-knee joint loading device delivers standardized and reproducible compressive loading of chondral samples. Distinct patterns of intra-tissue changes were detectable in morphology, sample height and pixel numbers, indicating effective sample pressurization. Loading-induced changes in qMRI parameters are reflective of sufficient sample pressurization. Consistent decreases in T1 were found in all samples, irrespective of degeneration, while significant increases in T1ρ and T2* were observed in *deg* samples only. For T2, changes were related to the tissue zone with decreases (superficial zone) and increases (deep zone) observed alike.

Loading-induced decreases in T1 have been reported before^13,19^, even though loading protocols differed. Xia *et al*. observed consistent reductions in T1 and a clear relation to the applied strain intensity^13^, which is reflected by our data as greater changes in T1 were found at δ_5.0_ than at δ_2.5_. Although reductions in T1 were observed throughout the entire tissue depth, superficial-zone changes were larger than deep-zone changes, which is due to marked structural and compositional differences in cartilage^23^: The superficial zone is softest because of its limited fixed charge density and water content^24^. As T1 is widely considered a marker of tissue hydration^25^, considerable water redistribution within and -most likely- out of the tissue happens with loading. Saarakkala *et al*. found that the water content hardly changes in early degeneration^9^; hence, similar loading-induced changes in T1, irrespective of degeneration, become plausible in light of the dominating effect of water on T1 characteristics^26^. Correspondingly, the diagnostic profile of T1 in differentiating *int* from *deg* cartilage is relatively weak as is demonstrated by only moderate sensitivity.

Disparate depth- and degeneration-dependent loading patterns were observed for T1ρ. Significant increases were found in the deep zone of *deg* samples only, although changes were similar, yet non-significant, in *int* samples. Opposite, yet non-significant changes were found in the superficial zone with T1ρ decreases in *int* samples and increases in *deg* samples. In absolute terms, changes in T1ρ were more pronounced in *deg* than *int* samples. These findings are in line with recent *in-vivo* data, as Souza *et al*. reported that changes in T1ρ are considerably larger in OA patients than controls^16^. However, they also observed significant decreases in the superficial and increases in the deep zone, irrespective of degeneration. Most likely, this discrepancy is secondary to doubtful patient allocation procedures based on radiographic evaluation, which is coarse and questionable when applied as reference measure^2^. Additionally, T1ρ changes of the medial compartment are not linearly corelated with overall OA severity^27^, thereby further compromising this reference measure.

Nonetheless, T1ρ is a promising indicator of biologically meaningful intra-tissue adaptive processes^12,15,16,18^ that may be visualized using serial T1ρ mapping, even though the exact determinant of T1ρ remains to be defined^2,4,28^.

Physiologically, these adaptive intra-tissue processes involve water redistribution and reductions in tissue thickness, thereby increasing the relative proteoglycan concentration. Additionally, the ECM is condensed, deformed and altered in its orientation so that, in conclusion, the complex interplay of the solid and fluid cartilage phases determines the tissue’s loading response in healthy and diseased cartilage^18^. The extent of these adaptive intra-tissue changes, however, is related to degeneration with larger changes noted in more degenerative tissue^9,10^.

Similar observations as for T1ρ were made for T2 with significant decreases in the superficial and increases in the deep zone. As T2 is an indicator of water content, collagen composition and collagen anisotropy^29^, these changes are also reflective of intra-tissue adaptations outlined above. The diagnostic profile of T2 is equally promising; yet, the discriminatory power of T2 and T1ρ in static and functional contexts is still discussed^12,17,30^ and remains to be defined in future *in-vivo* studies.

For T2*, significant loading-induced increases were only observed in *deg* samples. The exact structural and compositional correlate of T2* is still debated^31,32^; hence, the target structure and/or mechanism thus measured in its functional contribution still needs to be identified. Even though reports on whether biologically meaningful changes are detectable by T2* mapping are conflicting^7,31–33^, our study suggests that T2* can be successfully applied to assess degeneration-dependent adaptive intra-tissue changes in response to loading, in particular at large strains, which is in line with earlier findings^34^. However, multi-gradient echo sequences (used for T2* mapping) are more prone to susceptibility artefacts secondary to local magnetic field inhomogeneities^31–33^, specifically when interfaces (as in cartilage) are present. Also, T2* measurements are affected by refocusing pulses, gradient spoilers and echo spaces^32^, which limits inter-study comparability and questions the reliable inter-patient and inter-study quantification of T2* in cartilage, regardless of loading. Against this background, intra-patient referencing to the unloaded configuration as in our study seems to provide a scientifically sound and clinically feasible approach to eliminate inter-patient and inter-study concerns pertaining to variability in imaging protocols, unloading times, analysis routines, scanner and coil configurations, and methods of segmentation and registration^8^.

Nonetheless, this study suggests that cartilage functionality assessment ought to be multiparametric and serial T1ρ, T2 and T2* mapping techniques seem to be most promising to differentiate the tissue’s status in health and disease, not least due to their distinctly different sensitivity profiles^26^. If structural integrity of cartilage needs to be confirmed based on qMRI parameter values and their response-to-loading patterns, sensitivity needs to be highest and stand-alone T1ρ, T2 and the loading-induced changes in T2* should be assessed. Correspondingly, if degeneration needs to be diagnosed with a high degree of confidence, stand-alone T2 and the loading-induced changes in T2 should be studied.

Surprisingly, no significant correlations were found between relative changes in qMRI parameters and biomechanical or histological properties. Although significant differences in YM were found as a function of degeneration, the biomechanical properties are largely determined by ECM integrity rather than composition^35^. Therefore, compositional changes (as primarily assessed by MRI techniques) are not necessarily reflective of structural tissue properties.

Considerable standard deviations were observed throughout this study, which diminished statistical power. Biologically, the substantial inter-individual variability observed at δ_0_ is reflected by the samples’ variable loading responses. For the future *in-vivo* translation the predictive ability and diagnostic accuracy of these metrics have to be thoroughly addressed, especially in view of additional complexities such as joint positioning and compression status. Nonetheless, tissue degeneration is one contributory factor to cartilage functionality (among others) that needs to be considered alongside other patient- and joint-level factors such as age, sex, constitution and sports activities.

This study has limitations that involve technical, engineering and biological aspects. Considerable stress relaxation (of up to 48%)^22^ was observed during loading, which is not surprising, given the displacement-controlled loading setup. However, as the same order of sequence acquisitions (i.e. T2*-T2-T1ρ-T1) was maintained throughout the study, samples have experienced different loading conditions during T2* than T1 measurements. For improved standardisation, pressure-controlled loading configurations should be implemented. However, in this regard, further research is necessary to determine how exactly loading-induced changes in cartilage (as assessed by qMRI parameters) are related to stress in comparison to strain. Also, this setup’s realization of truly physiological exam conditions was limited as meniscus and ligaments were not included, which increases cartilage stress levels upon loading^36^. Additionally, any other physiological motion beyond uni-axial compression along the mechanical leg axis was precluded (i.e. the ‘screw-home’ mechanism at full extension). Loading therefore equalled a well-conformed, yet confined, compression test. Moreover, wirosil® (i.e. the artificial cartilage material) is slightly less stiff than human cartilage^22^ and future studies need to determine whether the observed response-to-loading patterns are similar to physiological cartilage-cartilage bearings. Yet, recent *in-vivo* data indicated that cartilage experiences a combination of compression and shearing when bearing weight^37^. As, physiologically, cartilage is relatively compliant, yet incompressible, and constrained by the underlying subchondral bone, compressive loading induces lateral expansion and secondary shearing, which is more relevant to the loading response than compression itself^37^.

Another important aspect is sample standardization. Chondral samples were cut to 3 mm thickness, which led to variability in the deeper tissue zones to be included per sample as cartilage thickness is variable. This is functionally relevant as deep tissue zones are essential for load-bearing due to their role in fluid convection and pressurization^38^. Hence, human cadaver or *in-patient* studies (with subsequent tissue harvest, e.g. response-to-loading assessment by MRI prior to total joint replacement) need to address these aspects to pave the technique’s clinical translation. In this context, standardization of loading of a given joint compartment or region and exact matching to reference measures are perspective challenges to tackle. Our sample source (i.e. joint replacement material) is problematic as even grossly intact samples exhibited signs of early degeneration such as matrix discoloration or hypercellularity, thereby rendering the dichotomization of *int* vs. *deg* samples somewhat arbitrary in view of the continuous degenerative changes. Additional tissue sources (i.e. organ donor networks or amputations) may help overcome this issue.

In conclusion, distinct patterns of qMRI parameter changes were found in this *in-situ* study and in response to loading. Changes in T1ρ, T2 and T2* were different in *deg* as compared to *int* cartilage, while changes in T1 were consistent in all samples irrespective of degeneration. Even though these metrics’ diagnostic accuracy and predictive ability needs to be better defined in entire joint configurations, the non-invasive assessment of cartilage functionality based on serial qMRI mapping provides an exciting framework to further stratify cartilage degeneration beyond mere static analysis.

## Methods

### Industry support

This study was supported by Philips Healthcare (Hamburg, Germany) by providing the T1ρ sequence. The authors had and have full control over the data and information submitted for publication.

### Study design

This study was designed as a prospective comparative *ex-vivo* imaging study of cartilage samples that were obtained from total knee replacements at our institution between 10/2015 and 10/2016. Local Institutional Review Board approval of all experimental protocols and the use of the cartilage-bone material (Ethical Committee, RWTH Aachen University, Germany, AZ-EK157/13) was obtained beforehand as well as individual informed patient consent. The methods were carried out in accordance with the relevant guidelines and regulations. This study’s datasets are available on sensible request by contacting the corresponding author.

### Compressive loading device

**Figure 5:**
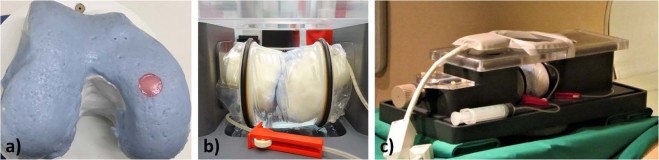
Device for the standardized, displacement-controlled compressive loading of human articular chondral samples. In preparation of this study, an intact right human knee joint had been scanned by CT to obtain the bone contours of femur and tibia which were 3D printed and subsequently covered by wirosil® silicone (as artificial cartilage layer) in its native configuration. (**a**) At the center of the medial femoral condyle a circular defect (8 mm diameter, 3 mm depth) was created within the wirosil® silicone (blue material) to be filled with native human articular cartilage samples of equal dimensions (red). (**b**) Loading was brought about by axial displacement of the tibial component (left) against the fixed femoral component (right). The standardized knee was covered by a transparent artificial joint capsule sealed with O-rings and filled with PBS buffer. (**c**) The assembled and sample-loaded device is displayed within the bore of a 3.0 T MRI system; for imaging, a dual-coil setup was used (attached above and below the knee).

The MRI-compatible whole-joint compressive loading device was described and validated in an earlier study^22^. Briefly, a right formalin-fixed human knee had been scanned by computed tomography in its native configuration and digitally processed to create standardized femoral and tibial bone models. The bone models had been covered by cartilage-mimicking polyvinyl-siloxane (Wirosil) as artificial femoral and tibial cartilage layers in their native configuration. A standardized defect at the central medial femoral condyle (8-mm diameter, 3-mm depth) had been created at the site of the initial contact into which the native chondral samples of corresponding dimensions were placed. Care was taken to precisely align the samples to avoid any step-offs (Fig. 5a). The standardized knee joint was surrounded by an artificial joint capsule filled with PBS buffer (Gibco-BRL) (Fig. 5b). Uni-axial compressive loading was performed by displacing the mobile tibia versus the immobile femur to 2.5 mm (δ_2.5_) and 5.0 mm (δ_5.0_) displacement (as measured from the initial contact point). Displacement-controlled loading resulted in mean forces on the entire joint as determined by use of a digital hydraulic force gauge (#HKMD29D, Induk, Wuppertal, Germany) of 141 ± 8 N (δ_2.5_) and 906 ± 38 N (δ_5.0_), corresponding to 20% and 110% of standard body weight. Mean local pressures on the chondral samples as determined by use of digital electronic pressure-sensitive sensors (K-Scan 4000, 10.000 psi, Tekscan, Boston, MA, USA) were 0.7 ± 0.1 MPa (δ_2.5_) and 1.1 ± 0.1 MPa (δ_5.0_). Of note, loading-induced strains within the chondral samples had not been determined. Compressive loading induced significant decreases in sample height (δ_0_: 2.86 ± 0.25 mm; δ_2.5_: 2.56 ± 0.25 mm; δ_5.0_: 2.02 ± 0.16 mm; p < 0.001 [repeated-measures ANOVA]), while sample circularity remained unchanged. Similarly, T2 signal intensity was decreased as a sign of sufficient tissue pressurization during loading. The interested reader may be referred to^22^ for additional information on the reference and validity measurements. MRI compatibility and the absence of significant B_0_-inhomogeneity was confirmed by B_0_-mapping.

### Cartilage sample preparation

Cartilage-bone material was harvested from 49 patients undergoing total knee arthroplasty at our institution (26 men, 23 women; 23 right and 26 left knees; mean age, 67.7 years [range, 46–93 years]) as before^19^. Primary OA of at least one joint compartment as determined radiographically (i.e. Kellgren-Lawrence grades ≥2^39^) was defined as the inclusion criterion, while all forms of secondary OA or other bone and joint disorders (e.g. avascular necrosis or rheumatoid arthritis) and a history of previous trauma or surgery to the knee were defined as exclusion criteria. After excision, the cartilage-bone material was collected in sterile Dulbecco’s modified Eagle’s medium (DMEM) containing 100 U/ml penicillin, 100 µg/ml gentamycin and 1.25 U/ml amphotericin-B (Gibco-BRL, Gaithersburg, US) and prepared according to standard: First, the medial femoral condyle was identified and only cartilage from that joint region was included for reasons of topoanatomic consistency. An 8-mm diameter skin biopsy punch and #13-scalpel (both from PFM-Medical, Cologne, Germany) were used to create cylindrical chondral samples by removing the subchondral bone. A dedicated metallic cutting device (i.e. metal block with circular moulds of 8-mm diameter and 3-mm depth) was used to cut chondral samples to standard thickness of 3 mm, which was confirmed using a standard digital micrometre (Mitutoyo-293-521, Tokyo, Japan). Second, samples were evaluated macroscopically by the first author (SN) according to the International Cartilage Repair Society (ICRS) classification^40^. Due to this study’s focus on differentiating intact from degenerative cartilage, only ICRS grades 0 (normal), 1 (superficial lesions) and 2 (fraying and lesions extending < 50% depth) were included, while more degenerative cartilage was discarded. Third, chondral samples were placed into the standard defect created within the artificial cartilage layer of the medial femoral condyle of the compressive loading device.

Based on earlier comparable studies^16,19^, sample size was estimated using a dedicated online tool (http://www.statstodo.com [Sample Size for Differences in Measurements Between Unpaired Groups Tables]). Minimum sample size of 34 was determined with the following parameters: statistical power 0.9, type-I-error 0.05, and effect size [mean of paired difference (to be detected)/expected standard deviation of paired difference] 0.8. To avoid sample pooling, one sample from each patient was harvested, i.e. a total of 49 samples.

### MRI measurements

After positioning the sample-loaded device centrally within a clinical 3.0 T MRI scanner (Achieva, Philips, Best, Netherlands), imaging was performed using standard general-purpose coils (Sense-Flex M, Philips) attached to the device’s upper and lower surfaces (Fig. 5c). The coils’ position was centered around the medial femoral condyle for maximized signal-to-noise ratio. MRI measurements were performed at three displacement positions as described above: a) unloaded (δ_0_), b) at δ_2.5_, and c) at δ_5.0_. Measurements at δ_0_ confirmed proper sample positioning and were used to determine reference qMRI parameter values. At each displacement position, stable sample position and proper displacement to δ_2.5_ and δ_5.0_ were confirmed using proton density-weighted (PDW) sequences acquired in the coronal, sagittal and axial plane (Fig. 2a–c). Sagittal and axial views were used to guide sections along the mid-coronal plane, thus creating a centrally bisecting section through the sample. Sequentially, T2*, T2, T1ρ and T1 sequences were obtained in this order; the sequence details are given in Table 4. Once the desired displacement position was set, an equilibration period of 5 min was observed prior to scanning. Using the inbuilt digital caliper tool of the picture archiving and communication system (PACS, Philips), the chondral sample’s height and width were determined at the sample center on mid-coronal PDW images. Measurements were undertaken at room temperature, which was monitored during the measurements (19.3 ± 0.4 °C).

**Table 4:** Acquisition Parameters of MR sequences.

	PDW	T1	T1ρ	T2	T2*
Sequence Type	Turbo spin echo	Inversion recovery	Spin-lock multi-gradient echo	Multi-spin echo	Multi-gradient echo
Repetition Time [ms]	4049	3000	30	1000	700
Ech time [ms]	15	7.2	3.1	n × 9.01 (n = 1–8)	2.84 + n × 4.54 (n = 0–9)
Turbo spin-echo factor	14	5	64	8	10
Field of view [mm]	160 × 160	30 × 30	30 × 30	30 × 30	30 × 30
Acquisition matrix	400 × 302	64 × 64	64 × 64	64 × 64	64 × 64
Reconstruction matrix	512 × 512	80 × 80	80 × 80	80 × 80	80 × 80
Flip angle [°]	90	90	11	90	55
Number of signal averages	1	2	4	3	8
Slices	28	1	1	1	1
Slice Thickness/Gap [mm]	3.0/3.3	2.0/n/a	3.2/n/a	2.0/n/a	2.0/n/a
Inversion times [ms]	n/a	150, 300, 500, 800, 1000, 1300, 1500	n/a	n/a	n/a
Spin-lock durations [ms]	n/a	n/a	0, 10, 20, 30, 40	n/a	n/a
Duration [min]	4.3	9.1	7.4	3.3	6.0

n/a - not applicable.

### MRI data extraction

After importing the MR raw data into Matlab R2016a software (Matlab, Natick, MA, US), respective time constants for each pixel of the mid-coronal image were calculated using predefined mono-exponential fitting routines as before^7,19^. Spatially resolved quantitative T1, T1ρ, T2, and T2* maps were generated for each sample and displacement position. For fitting, all values were included for the T1 and T1ρ maps, while the first echo and echo times >60 ms were disregarded for T2 and T2* because of potential fitting inaccuracies^41^ and too low signal-to-noise ratios. R² statistics adjusted to the degrees of freedom were used to check fit quality. Sample outlines were segmented manually based on the mid-coronal PDW image by choosing pixels that safely lay within the cartilage sample (Fig. 2d_1_). Correspondingly, boundary pixels (at the surface or bottom) were excluded to prevent partial volume effects. Segmented outlines were validated against T1, T1ρ, T2 and T2* maps. For zonal analysis, sample outlines were partitioned into two equal zones, i.e. the superficial (sf) and deep zone (dp), which were defined as the sample halves at the cartilage surface or subchondral bone, respectively (Fig. 2d_2–3_). Thus, the entire cartilage sample (ECS) with its cartilage zones (sf, dp) constituted the ROIs for which mean qMRI parameter values were calculated. For each sample and displacement position, ROIs were individually defined.

### Biomechanical analyses

After MRI measurements, the chondral sample was retrieved to undergo biomechanical testing as before^22^: unconfined compression, compression rate: 0.005%/s, maximum strain: 21%. A relatively low strain rate was chosen deliberately to assess the contribution of the ECM, which bears more than 80% of the applied load at a strain rate of 0.005%/s^42^. Load-displacement data were recorded and YM (defined as the stress-strain ratio) was determined by fitting a tangent to the strain range of 10–20%^43^. Throughout, samples were kept hydrated.

### Histological analyses

Chondral samples underwent standard histological assessment as before^7,19,22^. Additionally, macroscopically similar osteochondral tissue regions adjacent to the harvested chondral samples were also prepared to evaluate the cartilage-bone transition. Sections were prepared along the mid-coronal imaging plane or parallel to it. Following fixation in 4%-paraformaldehyde, samples were embedded in paraffin, cut to 5-µm sections, stained with hematoxylin/eosin and Safranin O and imaged using a standard light microscope (Leica-DM/LM-P, Wetzlar, Germany). Two investigators experienced in musculoskeletal histopathology (SN [9 years of experience], MP [3 years]) assessed the sections individually and graded them semi-quantitatively according to Mankin *et al*.^44^: Structure (score 0–6), cellularity (score 0–3), proteoglycan staining (score 0–4) and tidemark integrity (score 0–1) were scored and summed up (Mankin sum score [MSS]; range: 0–14). More severe degeneration is indicated by higher MSS. Structure, cells and proteoglycan staining were assessed on the chondral sections, while tidemark integrity was assessed on the adjacent osteochondral sections. If scores were different, sections were discussed until consensus. Subsequently, samples were dichotomized according to their MSS into intact (*int*, MSS 0–4) or early degenerative (*deg*, MSS 5–8) as in earlier comparable studies^45^.

### Statistical analyses

Statistical analyses were performed using GraphPad Prism (v6.0, San Diego, CA, US). As before^19,46^, qMRI data was log-transformed to achieve a Gaussian normal distribution, which was confirmed using the D’Agostino & Pearson’s test. Longitudinal differences were assessed using repeated measures ANOVA followed by Tukey’s multiple comparison test. Relative changes in qMRI parameters (i.e. Δ_2.5_, Δ_5.0_) were calculated on a per-sample basis by relating absolute values at δ_2.5_ and δ_5.0_ to δ_0_: Δ_2.5_ = ((δ_2.5_/δ_0_) − 1)*100 [%]; Δ_5.0_ = ((δ_5.0_/δ_0_) − 1)*100 [%], i.e. the means of the relative change per sample are reported. Two-tailed unpaired Student’s t-test was applied for group-wise comparisons, while correlations were quantified using Spearman’s correlation coefficient ρ. Data are presented as mean ± standard deviation or Spearman’s ρ (p-value). Level of significance was set to p ≤ 0.05 and further stratified into 0.01 < p ≤ 0.05 (*), 0.001 < **p ≤ 0.01 and ***p ≤ 0.001.

To assess the diagnostic performance of individual qMRI parameters, their relative changes and combinations in the differentiation of *int* and *deg* cartilage were classified as intact (i.e. belonging to the *int* group) or degenerative (i.e. belonging to the *deg* group) based on the respective qMRI parameter values. Threshold values for individual qMRI parameters were chosen based on the respective parameters’ ranges determined for *int* samples (Table 1): lower limit, M_int_ − SD_int_; upper limit, M_int_ + SD_int_. Scatter plots of data (i.e. qMRI parameter values vs. Mankin sum scores) were segregated into true positive, false positive, true negative, and false negative. The attribute true or false was determined by belonging to the *int* group as determined histologically, while the attribute positive or negative was determined by the respective qMRI parameter value. Once segregation was completed, the sensitivities and specificities of the respective qMRI parameters, their relative changes and several representative combinations was calculated. To maximize sensitivity, the *“Believe the positive”* rule was applied, i.e. a combined test is positive if either of the component tests is positive. To maximize specificity, the corresponding *“Believe the negative”* rule was applied^47^.

## Supplementary information

Supplementary Information

Supplementary Dataset KGA 4

## Data Availability

The datasets generated and analysed during the current study are available from the corresponding author on reasonable request.
