# Supplementary material for: Detection of Early-Stage Degeneration in Human Articular Cartilage by Multiparametric MR Imaging Mapping of Tissue Functionality
Source: Sci Rep. 2019 Apr 11;9:5895. doi: 10.1038/s41598-019-42543-w (PMC6459828; doi:10.1038/s41598-019-42543-w)
Supplement: Supplementary file 1 — Supplementary Information [file 41598_2019_42543_MOESM1_ESM.pdf]

Supplementary Material

Title: Detection of Early-Stage Degeneration in Human Articular Cartilage by Multiparametric MR Imaging Mapping of Tissue Functionality

Reference number / Journal: SREP-18-48715 / Scientific Reports

Authors: Sven Nebelung, Manuel Post, Matthias Knobe, Markus Tingart, Pieter Emans, Johannes Thüring, Christiane Kuhl, Daniel Truhn

Supplementary Figure S1

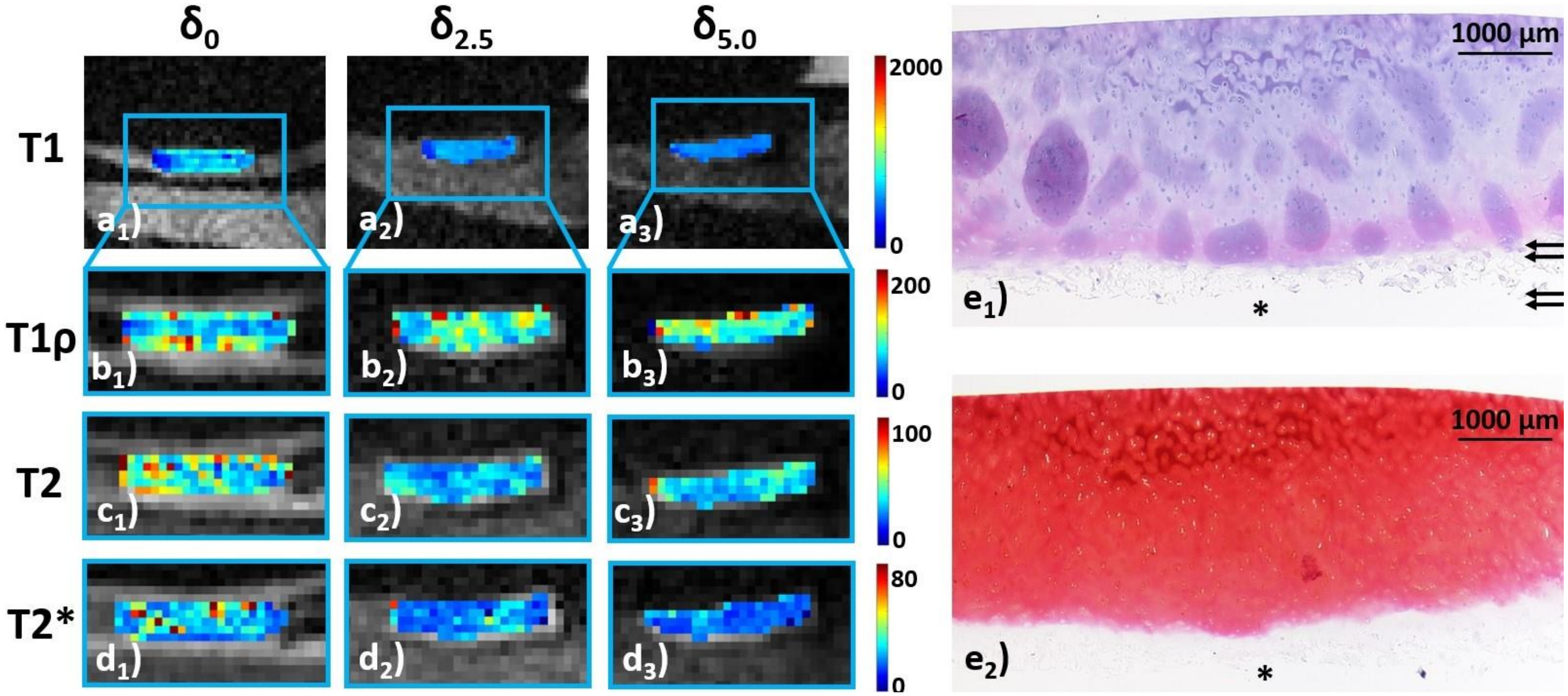

Supplementary Figure S1: Serial qMRI parameter maps under loading and corresponding histological sections of early degenerative cartilage.

Pre-existent focal signal hyperintensities became less distinct to nearly disappear altogether with loading. Histologically, pannus formation (indicated by vertical double arrows in e1) was noted alongside diffuse hypercellularity (only visible at higher magnification [not shown]) and moderate discoloration on proteoglycan staining. MSS 6. Image details as in Fig. 2.

Supplementary Table S1: Tukey's multiple comparison test details as outlined by consecutive numbers in Table 2. Asterisks mark significant differences ([***]: $p<0.001$; [**]: $0.001\leq p<0.01$; [*]: $0.01\leq p\leq 0.05$). 'int': histologically intact; 'deg': early degenerative; 'all': all samples; 'ECS': entire cartilage sample; 'sf': superficial zone; 'dp': deep zone.

	**T1 ECS all (1)**	**T1 sf all (2)**	**T1 dp all (3)**	**T1 ECS int (4)**	**T1 sf int (5)**	**T1 dp int (6)**	**T1 ECS deg (7)**	**T1 sf deg (8)**	**T1ρ dp all (9)**	**T1ρ dp deg (10)**	**T2 sf all (11)**	**T2 dp all (12)**	**T2 ECS int (13)**	**T2 sf int (14)**	**T2 dp int (15)**	**T2 dp deg (16)**	**T2* ECS deg (17)**
**p-value**	< 0.001	< 0.001	< 0.001	< 0.001	< 0.001	0.003	< 0.001	< 0.001	< 0.001	0.003	< 0.001	< 0.001	0.002	< 0.001	< 0.001	0.002	0.029
**δ_0 vs $\delta_{2.5}$**	***	***	ns	**	***	ns	**	***	**	*	ns	**	ns	ns	***	ns	ns
**δ_0 vs $\delta_{5.0}$**	***	***	***	***	***	**	***	***	**	**	***	***	ns	***	**	**	*
**$\delta_{2.5}$ vs $\delta_{5.0}$**	***	**	*	**	*	ns	ns	ns	ns	ns	***	ns	**	**	ns	*	ns

<u>Supplementary Table S2:</u> Relative changes of qMRI parameters in response to loading. Relative changes at 2.5 mm displacement ($\Delta_{2.5}$) and 5.0 mm displacement ($\Delta_{5.0}$) [%]. Data are M ± SD. Unpaired Student's t-test was used to detect group-wise differences between int and deg samples at each loading position. No significant group-wise differences in relative changes were found (p>0.05). Abbreviations as in Table 1.

	T1				T1ρ				T2				T2*			
	**$\Delta_{2.5}$ [%]**		**$\Delta_{5.0}$ [%]**		**$\Delta_{2.5}$ [%]**		**$\Delta_{5.0}$ [%]**		**$\Delta_{2.5}$ [%]**		**$\Delta_{5.0}$ [%]**		**$\Delta_{2.5}$ [%]**		**$\Delta_{5.0}$ [%]**	
	**int**	**deg**	**int**	**deg**	**int**	**deg**	**int**	**deg**	**int**	**deg**	**int**	**deg**	**int**	**deg**	**int**	**deg**
ECS	-7.6 ± 9.5	-6.0 ± 8.6	-13.2 ± 13.0	-9.9 ± 10.1	7.7 ± 25.6	9.6 ± 23.1	6.1 ± 26.4	15.6 ± 29.0	4.2 ± 8.4	1.5 ± 12.5	-2.7 ± 12.5	4.0 ± 14.4	3.5 ± 11.8	2.0 ± 13.3	2.0 ± 18.7	10.0 ± 18.4
sf	-12.0 ± 13.4	-9.4 ± 9.3	-18.0 ± 16.1	-13.6 ± 10.5	-0.1 ± 21.3	3.4 ± 25.8	-2.6 ± 25.3	10.0 ± 27.8	-0.8 ± 12.0	-0.3 ± 14.7	-10.1 ± 15.8	-4.5 ± 16.8	3.1 ± 15.9	11.0 ± 23.8	1.9 ± 22.5	11.9 ± 22.0
dp	-2.8 ± 6.4	-2.2 ± 11.3	-6.4 ± 13.3	-4.9 ± 12.7	14.8 ± 31.3	23.8 ± 31.1	16.5 ± 31.2	26.1 ± 37.9	10.4 ± 13.2	5.1 ± 15.0	7.9 ± 13.6	13.6 ± 17.3	3.0 ± 14.2	-5.4 ± 15.7	3.4 ± 19.7	5.1 ± 23.6

<u>Supplementary Table S3</u>: Spearman correlation coefficients between relative changes in qMRI parameters ($\Delta_{2.5}$ and $\Delta_{5.0}$) and biomechanical as well as histological parameters. Segmentation included the entire cartilage sample. Data are Spearman's correlation coefficient ρ with p-values in parentheses. No significant differences were found.

		**Young's Modulus**	**Mankin Sum Score**
**T1**	Δ2.5	0.207 (0.155)	0.010 (0.944)
	Δ5.0	0.164 (0.260)	0.220 (0.129)
**T1ρ**	Δ2.5	0.150 (0.375)	-0.095 (0.577)
	Δ5.0	-0.028 (0.867)	0.007 (0.967)
**T2**	Δ2.5	0.191 (0.188)	-0.129 (0.378)
	Δ5.0	-0.023 (0.874)	0.181 (0.215)
**T2***	Δ2.5	0.262 (0.069)	-0.036 (0.804)
	Δ5.0	0.135 (0.357)	0.213 (0.141)
